# Descending and Ascending Signals That Maintain Rhythmic Walking Pattern in Crickets

**DOI:** 10.3389/frobt.2021.625094

**Published:** 2021-03-29

**Authors:** Keisuke Naniwa, Hitoshi Aonuma

**Affiliations:** Research Institute for Electronic Science, Hokkaido University, Sapporo, Japan

**Keywords:** locomotion, rhythmic movement, cricket, gait, descending signal, ascending signal

## Abstract

The cricket is one of the model animals used to investigate the neuronal mechanisms underlying adaptive locomotion. An intact cricket walks mostly with a tripod gait, similar to other insects. The motor control center of the leg movements is located in the thoracic ganglia. In this study, we investigated the walking gait patterns of the crickets whose ventral nerve cords were surgically cut to gain an understanding of how the descending signals from the head ganglia and ascending signals from the abdominal nervous system into the thoracic ganglia mediate the initiation and coordination of the walking gait pattern. Crickets whose paired connectives between the brain and subesophageal ganglion (SEG) (circumesophageal connectives) were cut exhibited a tripod gait pattern. However, when one side of the circumesophageal connectives was cut, the crickets continued to turn in the opposite direction to the connective cut. Crickets whose paired connectives between the SEG and prothoracic ganglion were cut did not walk, whereas the crickets exhibited an ordinal tripod gait pattern when one side of the connectives was intact. Crickets whose paired connectives between the metathoracic ganglion and abdominal ganglia were cut initiated walking, although the gait was not a coordinated tripod pattern, whereas the crickets exhibited a tripod gait when one side of the connectives was intact. These results suggest that the brain plays an inhibitory role in initiating leg movements and that both the descending signals from the head ganglia and the ascending signals from the abdominal nervous system are important in initiating and coordinating insect walking gait patterns.

## Introduction

One of the common issues between biologists and robotics scientists is revealing the mechanisms underlying adaptive locomotion in animals. It is generally believed that insects appeared on the earth roughly 400 million years ago and that approximately 1,000,000 insect species are living on the earth. One of the reasons why insects have successfully evolved to spread across the earth may be the development of adaptive locomotion. Locomotion is the act of moving from place to place and is a crucial behavior for insects to obtain resources such as foods, territories, to find mating partners, to avoid predators, and so on. Revealing the neuronal mechanisms underlying locomotion in insects can aid in understanding the evolution of insect behaviors, as well as accelerate the development of novel design and control laws for legged robots.

This study focuses on cricket locomotion. Cricket is one of the ideal experimental animals to investigate neuronal mechanisms underlying varieties of behaviors such as locomotion [walking (Owaki et al., 2021), flight (Schul and Schulze, 2001; Pollack and Martins, 2007), swimming (Matsuura et al., 2002), aggressive behavior (Stevenson et al., 2000; Sakura et al., 2012; Rillich and Stevenson, 2014, 2017), escape behavior (Jacobs et al., 2008; Yono and Aonuma, 2008), mating behavior (Nagao et al., 1991; Ureshi et al., 2002; Nagamoto et al., 2005; Killian et al., 2006), learning and memory (Matsumoto et al., 2006), phonotaxis (Baden and Hedwig, 2008; Pollack and Kim, 2013), circadian rhythm (Saifullah and Tomioka, 2002) and so on]. On the other hand, in the robotics field, cricket inspired robots are made where design and control law of autonomous robots are investigated [the locomotion of micro-cricket robot: (Birch et al., 2000), phonotaxis robot: (Lund et al., 1997; Reeve et al., 2005), group behavior: (Funato et al., 2008, 2011), cricket-robot interaction: (Guerra et al., 2010; Kawabata et al., 2013a, Kawabata et al., 2013b)]. Some of the robotics scientists struggle to make hexapod robots that move like an insect (Delcomyn and Nelson, 2000; Meyer et al., 2020). However, probably because they employed centralized control, it seems hard to realize a robot that behaves adaptively like an insect. Other robotics scientists employ sensory-feedback-based control to realize adaptive locomotion (Owaki et al., 2017). But still, it seems difficult to realize exploratory behavior like an insect. To establish suitable design and control law for adaptive robots, it is one of the effective strategies to understand the adaptive behavior of insects using biological approaches.

Exploratory behavior to identify resources is initiated by the command signals generated in the brain. Thus, descending signals from the brain are necessary for the initiation of voluntary walking in both vertebrates and invertebrates (Kagaya and Takahata, 2011). External and internal signals are associated with the initiation of various behaviors. Chemical cues initiate exploratory behavior in insects because they are attracted by the chemical components of food and pheromones (Dethier, 1947). Auditory signals are another type of cue for attracting conspecific insects. For example, female crickets express phonotaxis to the calling song stridulated by males (Alexander, 1961; Nagao and Shimozawa, 1987; Jacob and Hedwig, 2016). Internal signals also function to initiate behaviors. Starvation and thirst can increase the motivation to initiate exploratory behavior for food and water, indicating that food digestion and the excretion system are associated with initiating behaviors in insects.

Insects are hexapod animals and most of them exhibit a tripod gait pattern, whereby the foreleg and hind leg on one side move in synchrony with the midleg on the other side (Wilson, 1966; Bender et al., 2011; Smolka et al., 2013; Ramdya et al., 2017). Descending signals via the central complex in the brain are important for initiating walking in insects (Strausfeld, 1999; Bender et al., 2010; Emanuel et al., 2020). The central complex is one of the important neuropils in the brain where multi kinds of sensory information are converged and processed, such as visual and olfactory information, auditory information (Homberg, 2008; Pfeiffer and Homberg, 2014). It is believed that the key role of the central complex is locomotor control (Strauss, 2002; Bender et al., 2010; Ritzmann et al., 2012), spatial orientation (Neuser et al., 2008; Triphan et al., 2010; Homberg et al., 2011), visual memory (Liu et al., 2006; Ofstad et al., 2011), and various forms of arousal (Lebestky et al., 2009; Kong et al., 2010). The local centers of the leg movements lie within the thoracic ganglia, where oscillatory neuronal activities, which are known as central pattern generators (CPGs), contribute to rhythmic leg movements (Borgmann et al., 2009). Descending information from the brain into the thoracic ganglia is necessary to coordinate the movement of the legs (Heinrich, 2002; Emanuel et al., 2020). The subesophageal ganglion (SEG) plays a crucial role in walking (Knebel et al., 2018). However, our previous study demonstrated that headless crickets do not exhibit voluntary walking, except following defecation (Naniwa et al., 2019). After-defecation walking is initiated by ascending signals from the terminal abdominal ganglion. This suggested to us that ascending signals from abdominal ganglia may also contribute to coordinated walking. Indeed, cricket elicits avoidance walk by responding to air displacement that is detected by circus (Camhi et al., 1978; Shimozawa et al., 2003; Dupuy et al., 2011). The sensory signals from the circus are converged and processed in the terminal abdominal ganglion (Kanou and Shimozawa, 1984; Yono and Aonuma, 2008). Activation of ascending giant interneurons introduce activation of motor control in the thoracic ganglia to initiate avoidance walk (Ritzmann and Camhi, 1978; Ritzmann and Pollack, 1986). It is also demonstrated that abdominal ganglia in the cricket control the timing of the calling song pattern (Jacob and Hedwig, 2016). These indicate that the ascending signals from the abdominal ganglia can mediate neuronal activities of the thoracic ganglia. Thus, understanding the roles of ascending signals from the abdominal ganglia must be necessary to reveal the neuronal mechanism underlying adaptive locomotion in insects.

In this study, we aimed to determine how the ascending signals from the abdominal nervous system and the descending signals from the brain and SEG influence the coordinated walking gait pattern. To investigate this issue, we surgically cut the connectives of the ventral nerve cord at different positions and analyzed the walking gait pattern of the field cricket. To determine the roles of the brain in initiating and regulating the walking gait, either the paired connectives or one side of the connectives between the brain and SEG were cut. To investigate the roles of the SEG, either the paired connectives or one side of the connectives between the SEG and prothoracic ganglion were cut. Furthermore, to investigate the roles of the ascending signals from the abdominal nervous system, either the paired connectives or one side of the connectives between the metathoracic ganglion and first free abdominal ganglion were cut. Based on these results, we demonstrated that both the descending signals and the ascending signals into the thoracic ganglia play an important role in maintaining a coordinated walking pattern.

## Materials and Methods

### Animals

The cricket *Gryllus bimaculatus* (De Geer) used in this study were raised in a laboratory colony. They were reared on a 14 h:10 h light and dark cycle (lights on at 6:00 h) at 28 ± 2°C. They were fed a diet of insect food (Sankyo Lab, Tokyo, Japan) and water *ad-libitum*. Adult male crickets that had molted within 2 weeks before the experiments were randomly selected for use in this study.

### Behavioral Experiments

The crickets used were randomly selected from the colony. A cricket was placed on a handmade passive treadmill using a floating ball to observe its walking pattern. The treadmill ball was composed of a Styrofoam sphere (ϕ150 mm) that hovered over a stream of air flowing beneath it. Each cricket was anesthetized with CO_2_ gas for 10 s and was then placed on the ball. A steel rod (ϕ100 μm) was attached to the thorax of the cricket using dental wax (Shofu, Kyoto, Japan). The rod was inserted into a plastic tube (ϕ500 μm) that was fixed to a manipulator, by means of which the cricket was placed in the exact desired position on the Styrofoam sphere (floating ball). The behavioral experiment was performed 1 h after the cricket was placed on the ball so that it adapted to the new circumstances. A cricket on the ball could walk as well as change its orientation and ground clearance freely.

To investigate the roles of either the ascending or descending signals into the thoracic ganglia, where the premotor signals for locomotion are generated, the intersegmental connectives between the brain and SEG, between the SEG and prothoracic ganglion, and between the metathoracic ganglion and abdominal ganglia were cut using a razor blade. Surgical treatment was performed after administering anesthesia. A cricket held by hand was placed under the dissection microscope (SZX-12, Olympus, Tokyo Japan), and then a small square window was opened on the head to cut the intersegmental connectives. The cuticle cut off was replaced, and the hemolymph clotted quickly to close the window. Behavioral experiments were performed 3 h after surgical treatment.

The locomotion patterns of the crickets were observed and recorded using a high-speed camera (800 × 600 pixels, 300 fps, HAS-L1, DITECT, Japan). Intact crickets and the crickets whose intersegmental connectives between thoracic ganglia and abdominal ganglia were cut initiated voluntary walking on the ball. In contrast, the cricket whose paired circumesophageal connectives were cut did not walk without external stimulation. To initiate walking, the cercus of the cricket was stimulated by touching with a paintbrush. The detail of the touching stimuli is described in the previous study (Aonuma, 2020). Tactile stimuli were applied once or twice using a paintbrush in each trial and intertrial interval was varied between 1 and 5 min to prevent habituation. Continuous walking-period was shortened in connective-cut crickets compared to intact one (see Table 1). Therefore, we focused on a continuous walking-period to analyze gait patterns. The images were saved as sequential JPEG files on a Windows PC for subsequent analysis.

**Table 1:** Summary of the results.

**Specimens**	**Number of specimens**	**Average time analyzed [sec]**	**Average number of steps analyzed ± SD[step]**	**Combination of legs**	**Sample size: total number of steps analyzed [step]**	**Φ [deg]**	**ν [deg]**	***R***	***d***	**Power**	**Kuiper test vs. intact** **Kuiper statistic**	**V-test for 180** **V Statistic**
(A)	*N* = 5	3.06	11.3 ± 3.0	LF-RF	113	185	39.6	0.79			-	9.97^†^ *P* < 0.01
				LM-RM	113	165	47.6	0.71			-	9.00^†^ *P* < 0.01
				LH-RH	114	180	28.9	0.88			-	12.1^†^ *P* < 0.01
				LF-LM	112	212	42.2	0.76			-	8.98^†^ *P* < 0.01
				LM-LH	113	225	42.6	0.76			-	6.76^†^ *P* < 0.01
				LF-LH	114	68.7	62.1	0.56			-	−2.50 *P* = 0.99
				RF-RM	114	194	35.1	0.83			-	9.96^†^ *P* < 0.01
				RM-RH	114	241	29.3	0.88			-	4.14^†^ *P* < 0.01
				RF-RH	114	71.7	57.9	0.60			-	−2.45 *P* = 0.99
(B)	*N* = 5	2.46	5.8 ± 2.3	LM-RM	57	193	55.5	0.63	0.54	0.90	0.18 *P* = 0.87	5.45^†^ *P* < 0.01
(C)	*N* = 5	3.30	6.2 ± 2.6	LM-RM	57	187	131	0.07	0.22	0.27	0.57 *P* < 0.01	−2.12 *P* = 0.98
(D)	*N* = 5	11.5	0.7 ± 0.6	LM-RM	-	-	-	-	-	-	-	-
(E)	*N* = 5	2.86	11.1 ± 3.5	LM-RM	109	165	37.3	0.81	0.00	0.05	0.23 *P* = 0.56	9.53^†^ *P* < 0.01
(F)	*N* = 5	8.47	12.2 ± 3.7	LM-RM	121	109	108	0.17	0.67	0.99	0.59 *P* < 0.01	−1.38 *P* = 0.92
(G)	*N* = 5	6.43	6.4 ± 2.7	LM-RM	63	87.5	110	0.16	0.91	1.00	0.58 *P* < 0.01	−3.46 *P* = 1.00
(H)	*N* = 5	3.26	7.6 ± 2.7	LM-RM	71	199	108	0.17	0.41	0.74	0.41 *P* < 0.01	1.65 *P* = 0.05
(I)	*N* =5	1.71	4.1 ± 1.5	LM-RM	51	180	55.7	0.62	0.29	0.38	0.23 *P* = 0.56	4.12^†^ *p* < 0.01

***(A)** Intact crickets. **(B)** Crickets whose paired circumesophageal connectives were cut. **(C)** Crickets whose left side of the circumesophageal connective were cut. **(D)** Crickets whose paired connectives between SEG and prothoracic ganglion were cut. **(E)** Crickets whose left side of the connective between SEG and prothoracic ganglion was cut. **(F)** Crickets whose left side connective between brain and SEG, and left side connective between SEG and prothoracic ganglion were cut. **(G)** Crickets whose right side connectives between brain and SEG, and left side connectives between SEG and prothoracic ganglion were cut. **(H)** Crickets whose paired connectives between metathoracic ganglion and 1st free abdominal ganglion were cut. **(I)** Crickets whose left side connective between metathoracic ganglion and 1st free abdominal ganglion was cut. Φ, mean phase difference; ν, circular standard deviation; R, mean resultant length; d, effect size. Kuiper statistic is a descriptive statistic for a two-sample Kuiper test with intact crickets. The significance level α = 0.05; V statistic is a descriptive statistic based on the V-test for 180. It tests the null hypothesis that there is no tendency for leg phase differences to be distributed around 180. Significance level α = 0.05*.

† *the V statistic is greater than the rejection threshold at α = 0.05 (Batschelet, 1981)*.

### Data Analysis

To analyze and evaluate the leg movement patterns, we drew polar histograms (Naniwa et al., 2020), in which we focused on the leg movement direction. In brief, we defined the power stroke as the thrust produced when the angle between the femur and tibia increased in the case of the hindleg, or when the angle between the femur and trunk increased in the case of the foreleg and midleg. During the recovery stroke, the angle between the femur and tibia decreased for the hindleg or the angle between the femur and trunk decreased for the foreleg and midleg. The stroke mode was obtained manually from the video data. The condition of each leg in a frame was compared to those of the adjacent frames to determine whether it was a power or recovery stroke.

In the definition of the phase for each leg, *t* is a certain time and *t*_*n*_ is the start time of the power stroke directly before the *n*th step of the leg of interest.

The phase ϕ at a time *t* is defined as

$$\begin{array}{l} \phi \left(\text{t}\right)=\frac{\text{t}-{\text{t}}_{\text{n}}}{{\text{t}}_{\text{n}+1}-{\text{t}}_{\text{n}}}360\left(\text{deg}\right). \end{array}$$

Therefore, the leg phase is defined as the period between the beginning of two consecutive power strokes. In this case, ϕ_object_, ϕ_subject_ are the phases of an arbitrary leg, where the subscripts object and subject indicate the leg positions (e.g., LF, RM).

The leg phase difference of the subject leg relative to the object leg at a time *t* is expressed as

$$\begin{array}{l} {\bar{\phi }}_{\text{object-subject}}\left(\text{t}\right)=\\ \;\{\begin{array}{c} \phi_{\text{object}}\left(\text{t}\right)-\phi_{\text{subject}}\left(\text{t}\right)\;\left(\phi_{\text{object}}\geq \phi_{\text{subject}}\right)\\ \phi_{\text{object}}\left(\text{t}\right)-\phi_{\text{subject}}\left(\text{t}\right)+360\left(\phi_{\text{object}}<\phi_{\text{subject}}\right) \end{array}. \end{array}$$

This method aims to provide an intuitive and precise representation of the rhythmic pattern corresponding to the variations in the cricket legs owing to movement. Therefore, even in a polar representation, in which the area represents the ratio of frequencies, the height of a bar is the value of the square root of the frequency that it represents. As a result, the total area of the bar is 1 in a polar histogram (Nemec, 1988). The phase difference between the legs can be calculated for each frame. The polar histogram of the experimental results represents a summary of the frequencies of leg phases for all individuals and all frames in each experimental pattern. In an ideal tripod, the leg phase difference between adjacent legs (e.g., LF and RF or LF and LM) is always 180°.

In the polar histogram, the phase mean Φ is calculated as:

$$\begin{array}{l} R{\text{e}}^{i\phi }\text{=}\frac{\text{1}}{N}\sum_{\text{t}}{\text{e}}^{i\bar{\phi }\left(\text{t}\right)}, \end{array}$$

where *R* is the mean resultant length of each histogram, *N* is the total amount of sample data, and *i* is an imaginary number.

The circumferential dispersion *s* and circumferential standard deviation ν of the circumferential data are defined as follows:

$$\begin{array}{l} \text{s}\equiv 1-R\;\left(0\leq s\leq 1\right)\\ \nu \equiv \sqrt{-2\log\left(R\right)}. \end{array}$$

The rank statistics of the measured circumference data $\overset{-}{\phi }\left(t\right)$, sorted in ascending order in the range of 0 ≤ ϕ < 2π, are represented by $\left\{{\overset{-}{\phi }}_{\left(1\right)}^{*},{\overset{-}{\phi }}_{\left(2\right)}^{*},\cdots \;,{\overset{-}{\phi }}_{\left(N\;\right)}^{*}\right\}.$

In this case, the empirical distribution function *S*(ϕ) can be expressed as

$$\begin{array}{l} \text{S}\left({\bar{\phi }}_{\left(n\right)}^{*}\right)=n/N,\;n=1,2,\cdots \;N. \end{array}$$

The variations in the phase differences between legs could be intuitively understood by comparing the shapes of the empirical distribution functions. In this study, the empirical distribution function of the leg phase difference between the midlegs in each experiment is illustrated as a representative example. The phase distribution of the midlegs was tested. G^*^Power (Version 3.1.9.6) was used to conduct a *post-hoc* analysis of effect size *d* and power—the significance level α =0.05. The two-sample Kuiper test was performed for comparison with midleg phase distribution of intact cricket. The two-sample Kuiper test assesses the anomaly of continuous, one-dimensional probability distributions (Kuiper, 1960; Paltani, 2004). The V-test was performed to confirm that the midleg phases were in the opposite phase. It tests the null hypothesis that there is no tendency for leg phase differences to be distributed around 180. The number of specimens used in each experimental condition was five. The samples used for the tests were the leg phase at the timing of each leg grounding (*n* = 51–121).

## Results

**Figure 1:**
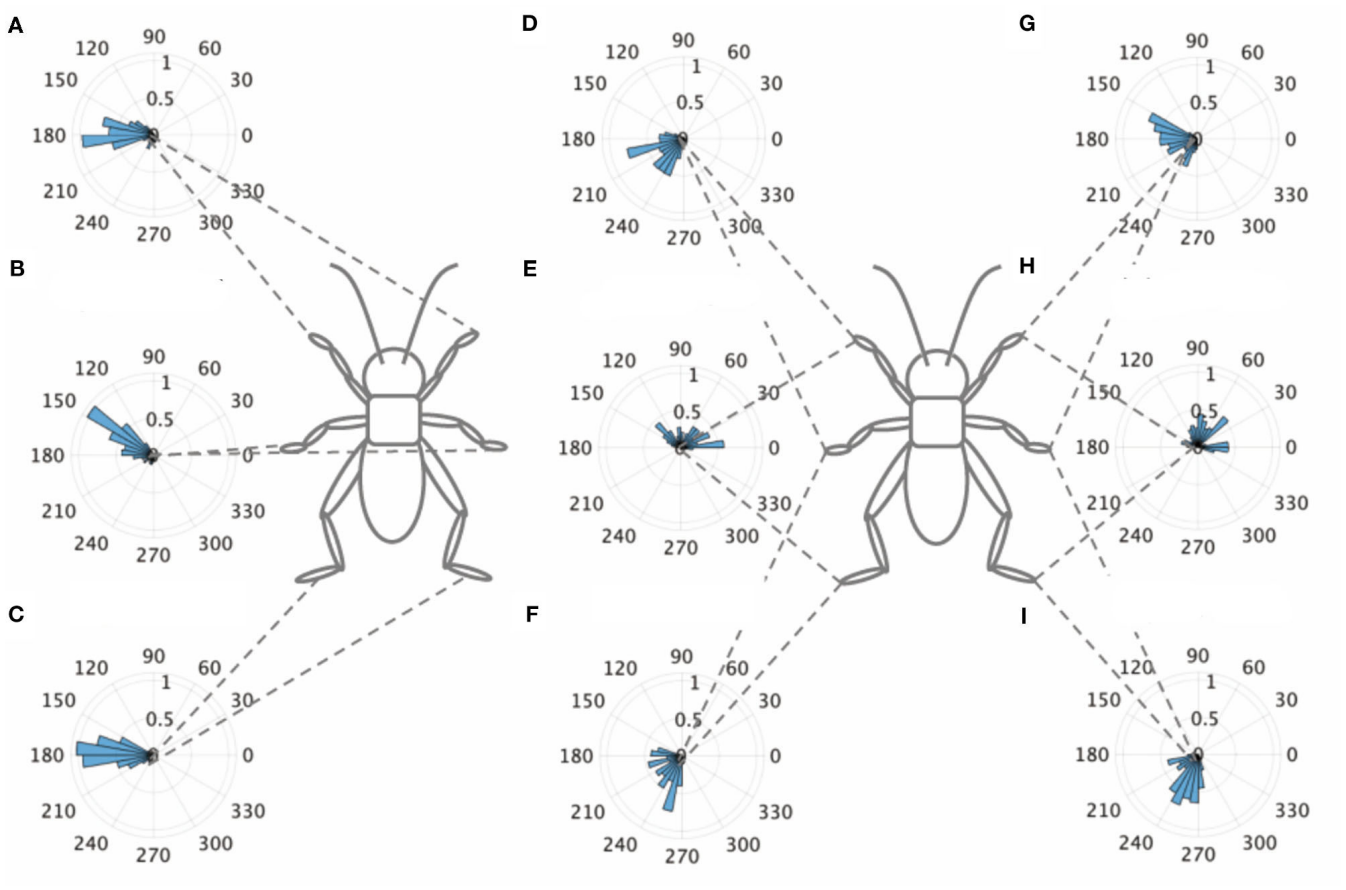
Walking gait patterns of intact crickets. Polar histograms indicate the phase differences between two legs in the intact crickets (*N* = 5), where the radial axis is the probability that the intact crickets exhibited a tripod gait pattern on the floating ball of the treadmill. **(A)** The phase difference between left and right forelegs. **(B)** The phase difference between left and right midlegs. **(C)** The phase difference between left and right hindlegs. **(D)** The phase difference between left foreleg and midleg. **(E)** The phase difference between left foreleg and hindleg. **(F)** The phase difference between left midleg and hindleg. **(G)** The phase difference between right foreleg and midleg. **(H)** The phase difference between right foreleg and hindleg. **(I)** The phase difference between left midleg and hindleg. LF, left foreleg; RF, right foreleg; LM, left midleg; RM, right midleg; LH, left hind leg; RH, right hind leg.

An intact cricket was anesthetized and placed on the floating ball of the treadmill. After recovered from anesthesia, voluntary evoked walking of the cricket was observed and recorded for 10 min, and then the periods of continuously walking were focused to analyze the gait pattern. The intact crickets exhibited a tripod gait pattern during walking on the floating ball of the treadmill (*N* = 5, Table 1A, Figure 1, Supplementary Video 1). The polar histogram indicates the phase difference between two of the six legs. The phase difference between the left and right forelegs occurred in an almost anti-phase manner. The mean of the foreleg phase difference Φ_*LF*−*RF*_ was 185°, with a standard deviation ν_*LF*−*RF*_ of 39.6°. The mean vector length *R*_*LF*−*RF*_ was 0.79. Similarly, the left and right midlegs moved in an anti-phase manner. The mean of the midleg phase difference Φ_*LM*−*RM*_ was 164°, with a standard deviation ν_*LM*−*RM*_ of 47.6°. The mean vector length *R*_*LM*−*RM*_ was 0.71. The left and right hindlegs also moved in an anti-phase manner. The mean of the midleg phase difference Φ_*LH*−*RH*_ was 180°, with a standard deviation ν_*LH*−*RH*_ of 28.9°. The mean vector length *R*_*LH*−*RH*_ was 0.88. The foreleg and midleg on the same side moved in an almost anti-phase manner (Φ_*LF*−*LM*_:212, ν_*LF*−*LM*_:42.2, *R*_*LF*−*LM*_:0.76, Φ_*RF*−*RM*_:194, ν_*RF*−*RM*_:35.1, *R*_*RF*−*RM*_:0.83), and the foreleg and hind leg on the same side moved slightly later than in-phase (Φ_*LF*−*LH*_:68.7, ν_*LF*−*LM*_:62.1, *R*_*LF*−*LM*_:0.56, Φ_*RF*−*RH*_:71.7, ν_*RF*−*RH*_:57.9, *R*_*RF*−*RH*_:0.60). A V-test for 180 was also performed on the inter-leg phase differences of the intact crickets. The leg phases tended to be concentrated at 180°, between the adjacent legs in the tripod gait [LF-RF: *n* = 113, V statistic for 180°: 9.97, LM-RM: *n* = 113, V statistic for 180°: 9.00 (*P* < 0.01), LH-RH: *n* = 114, V statistic for 180°: 12.1, LF-LM: *n* = 112, V statistic for 180°: 8.98 (*P* < 0.01), LM-LH: *n* = 113, V statistic for 180°: 6.76 (*P* < 0.01), RF-RM: *n* = 114, V statistic for 180°: 9.96 (*P* < 0.01), RM-RH: *n* = 114, V statistic for 180°: 4.14 (*P* < 0.01), Table 1A]. However, the degree of concentration varied. These results indicate that the legs did not maintain a perfectly coordinated relationship with one another during the tripod gait in the intact crickets. The intact crickets maintained the leg-phase relationship characteristic of a tri-pod gait with a certain degree of variability.

To investigate the manner in which the ordinary tripod gait pattern is regulated by descending signals from the brain or ascending signals from the abdominal nervous system, the connectives of the ventral nerve cord were surgically disconnected. The central nervous system of insects has a symmetric structure. The brain (protocerebrum, deutocerebrum, and tritocerebrum) is joined by paired nerve connectives to the SEG, which is, in turn, linked to the thoracic and abdominal ganglia by paired connectives.

### Disconnection of Circumesophageal Connectives

**Figure 2:**
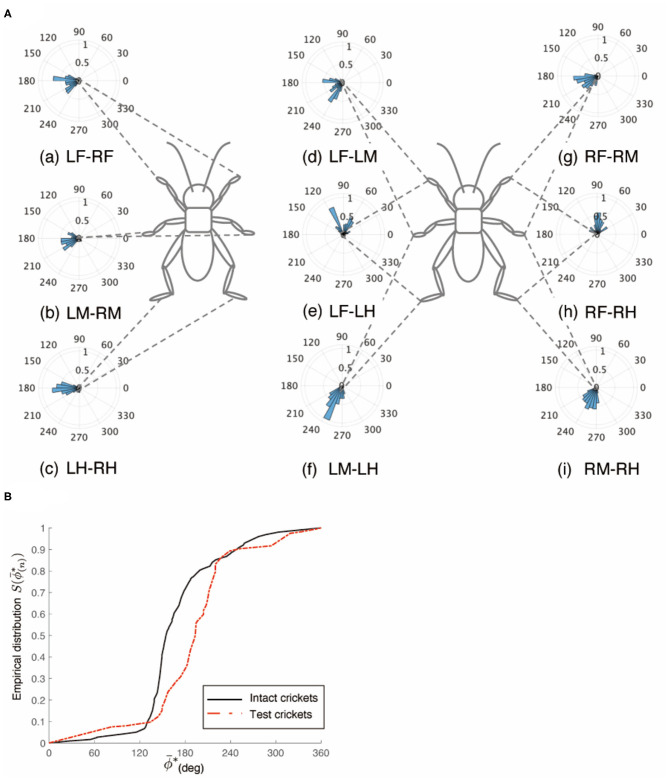
Walking gait pattern of crickets in which paired nerve connectives between brain and SEG were cut. **(A)** Polar histograms indicate phase differences between two legs in test crickets (*N* = 5). The polar histograms demonstrate that the test crickets exhibited a tripod gait pattern on the floating ball of the treadmill. (a–i) Phase differences between pairs of legs. **(B)** Comparison of the empirical distribution function of leg phase differences between left and right legs Φ_*LM*−*RM*_ The black line indicates the empirical distribution function of the intact crickets and the red line indicates that of the crickets in which the pair of connectives between the brain and SEG was cut. LF, left foreleg; RF, right foreleg; LM, left midleg; RM, right midleg; LH, left hind leg; RH, right hind leg.

The cricket whose paired circumesophageal connectives were cut did not show voluntary evoked walking. To investigate the walking gait pattern of the surgically treated cricket, we touched the cercus using a fine paintbrush to evoke walking. The disconnection of the paired circumesophageal connectives did not change the walking gait pattern of the test crickets, which walked on the floating ball with a tripod gait (*N* = 5, Figure 2A, Supplementary Video 2). The test crickets did not respond to tactile stimuli on the antennae, although they responded to tactile stimuli on the cercus while walking. This indicates that the descending signals from the brain into the SEG were shut down. The crickets mainly walked straight forward and did not turn voluntarily. The phase difference between the left and right midlegs occurred in an anti-phase manner (Figure 2Ab). In the intact crickets, the mean midleg phase difference Φ_*LM*−*RM*_ was 164°, with a standard deviation ν_*LM*−*RM*_ of 47.6°. The mean vector length *R*_*LM*−*RM*_ was 0.71. In contrast, in the test crickets, the mean of the midleg phase difference Φ_*LM*−*RM*_ was 193°, with a standard deviation ν_*LM*−*RM*_ of 55.5°. The mean vector length *R*_*LM*−*RM*_ was 0.63. The shape of the empirical distribution function of the midlegs of the test crickets was similar to that of the intact crickets (Figure 2B). The inter-leg phase difference of the midlegs of the test cricket was not significantly different from that of intact cricket and was concentrated in an anti-phase manner [LM-RM: *n* = 57, Kuiper statistic vs. intact LM-RM:0.18 (*P* = 0.87), V statistic for 180°: 5.45 (*P* < 0.01), Table 1B]. In contrast, leg frequencies tended to be lower than those of intact crickets (Supplementary Figure 1). This indicates that the gait pattern of the test cricket is classified as a tripod gait although its walking pattern is slightly different from that of the intact cricket.

**Figure 3:**
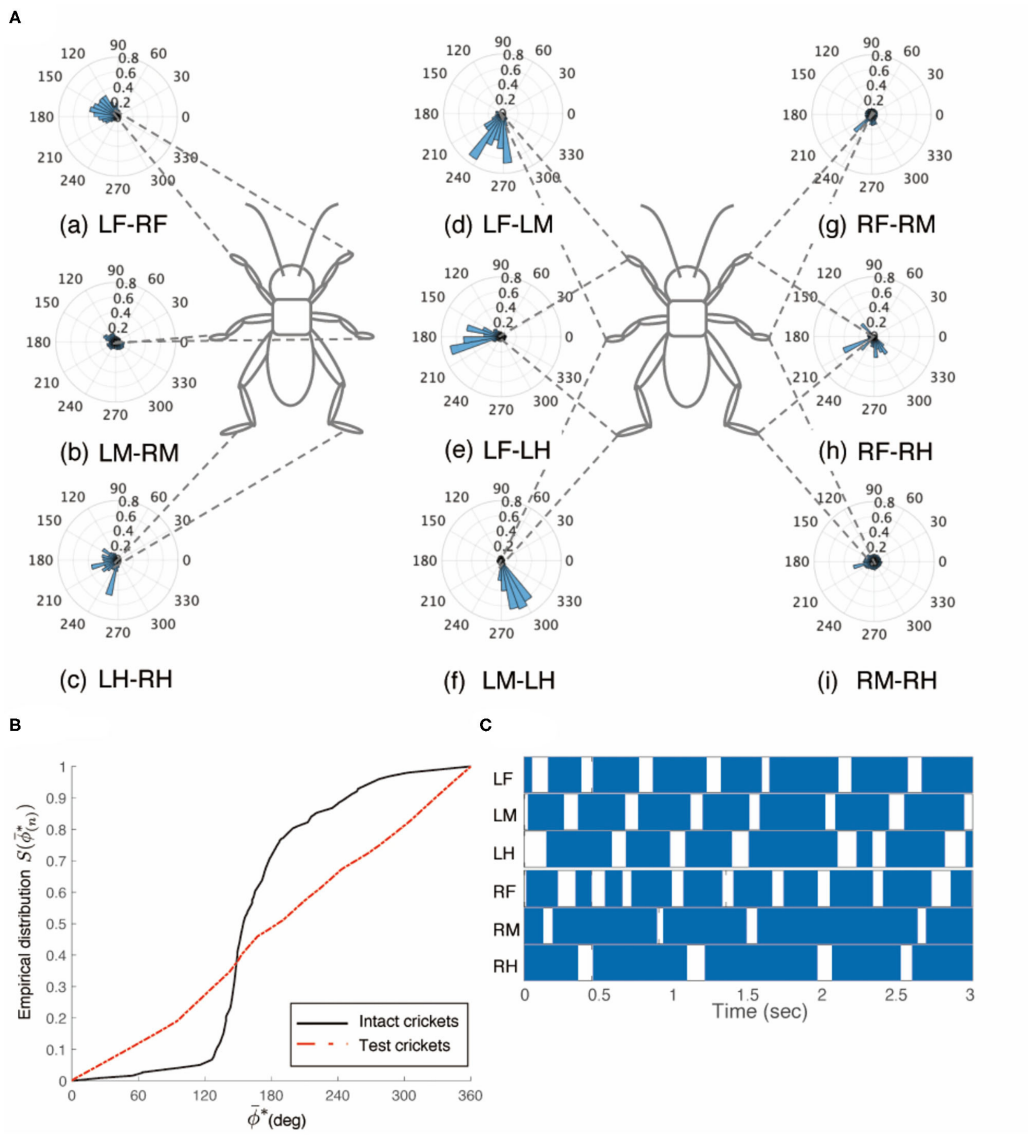
Walking gait patterns of crickets in which left side of nerve connectives between brain and SEG was cut. **(A)** Polar histograms indicate phase differences between two legs in test crickets (*N* = 5). The test crickets continued to turn clockwise. The polar histograms demonstrate that the walking pattern was not a tripod gait. (a–i) Phase differences between pairs of legs. **(B)** Comparison of the empirical distribution function of leg phase differences between left and right legs Φ_*LM*−*RM*_. The black line indicates the empirical distribution function of the intact crickets and the red line indicates that of the test crickets. **(C)** Gait chart diagram of test cricket. The filled part indicates the duration of the power stroke period and the blank part indicates the duration of the recovery stroke. This also demonstrates that the walking pattern was not a tripod gait in the test cricket. LF, left foreleg; RF, right foreleg; LM, left midleg; RM, right midleg; LH, left hind leg; RH, right hind leg.

However, the crickets in which only the left side of the circumesophageal connectives was cut did not walk straight forward but continued to turn clockwise (Supplementary Video 3). This kind of surgically treated crickets showed voluntary evoked walking without tactile stimuli. Their gaits did not exhibit an ordinary tripod pattern (*N* = 5, Figure 3). The polar histogram of these test crickets indicates that the phase differences between the left and right legs were not consistent (Figure 3A). In the test crickets, the mean of the midleg phase difference Φ_*LM*−*RM*_ was 187°, with a standard deviation ν_*LM*−*RM*_ of 131° (Table 1C). The mean vector length *R*_*LM*−*RM*_ was 0.07. The shape of the empirical distribution function of the midlegs in the test crickets was different from that of the intact crickets [LM-RM: *n* = 57, Kuiper statistic vs. intact LM-RM:0.57 (*P* < 0.01), V statistic for 180°: −2.12 (*P* = 0.98), Table 1C, Figure 3B]. This analysis also demonstrates that the walking pattern was far from the ordinary tripod gait (Figure 3C). The gait chart diagram of the test crickets reveals that the duration of the left leg movements appeared to be rhythmic, similar to that of the intact crickets. The duration of the right legs touching the floor was much longer than that of the left legs. The frequency of the right legs was lower than that of the left side, and the stroke angle of the right legs was smaller than that of the left side (Supplementary Figure 1C). This indicates that the left legs moved more than the right legs, making the cricket continue to turn clockwise. Similarly, when the right side of the circumesophageal connective was cut, the test crickets continued to turn counterclockwise and did not exhibit a tripod walking gait pattern (Supplementary Video 4).

### Disconnection of Connectives Between SEG and Prothoracic Ganglion

**Figure 4:**
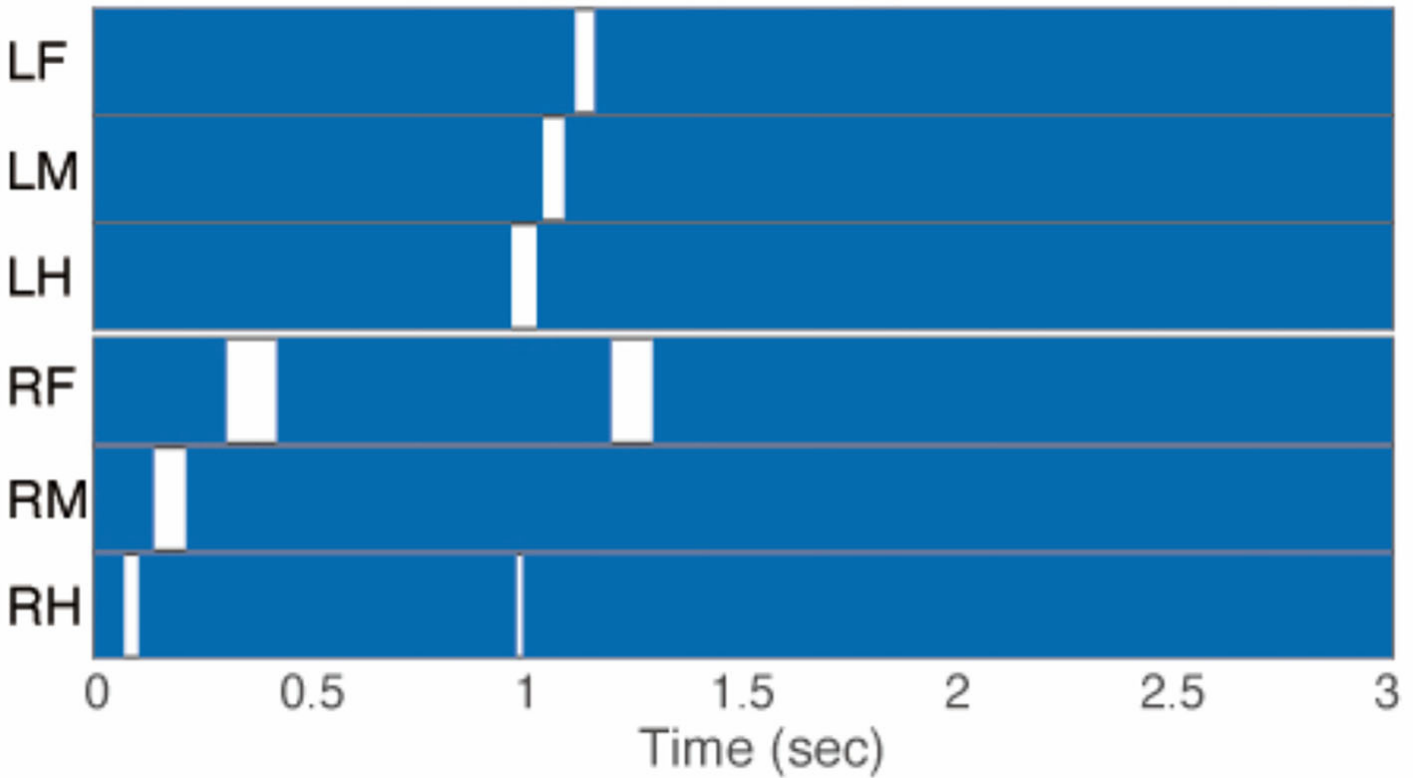
Gait chart diagram of cricket in which paired nerve connectives between SEG and prothoracic ganglion were cut. The filled parts indicate that the tip of the legs touched the floor, demonstrating that the cricket did not walk on the floating ball of the treadmill. LF, left foreleg; RF, right foreleg; LM, left midleg; RM, right midleg; LH, left hind leg; RH, right hind leg.

**Figure 5:**
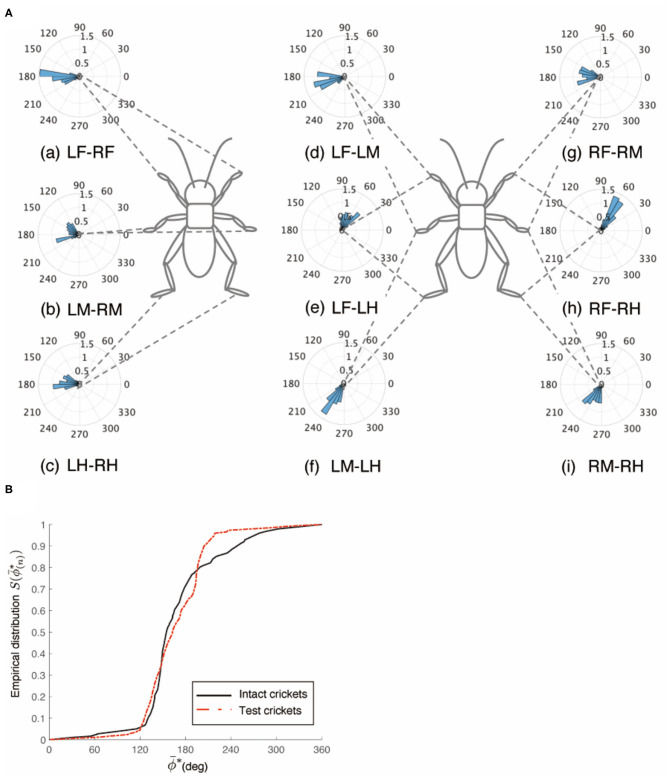
Walking gait patterns of crickets in which left side of nerve connectives between SEG and prothoracic ganglion was cut. **(A)** Polar histograms indicate phase differences between two legs in test crickets (*N* = 5). The polar histograms demonstrate that the test crickets exhibited a tripod gait pattern on the floating ball of the treadmill. (a–i) Phase differences between pairs of legs. **(B)** Comparison of the empirical distribution function of leg phase differences between left and right legs Φ_*LM*−*RM*_. The black line indicates the empirical distribution function of the intact crickets and the red line indicates that of the crickets in which only the left side of the nerve connectives between the metathoracic ganglion and abdominal ganglia was cut. LF, left foreleg; RF, right foreleg; LM, left midleg; RM, right midleg; LH, left hind leg; RH, right hind leg.

To investigate the role of the SEG, the paired connectives between SEG and prothoracic ganglion in the crickets were surgically cut. The behavior of these test crickets was the same as those of the headless crickets previously reported (Naniwa et al., 2019). The test crickets did not show voluntary evoked walking on the ball, except during defecation. They did not respond with walking to the tactile stimuli using the paintbrush. Therefore, the gait chart diagrams indicate that all legs of the crickets were always on the ground (*N* = 5, Figure 4, Supplementary Video 5). All test crickets did not walk a sufficient number of steps to analyze leg phase difference, frequency, and amplitude (average *n* = 0.7, Table 1D). However, if only one of the connectives between the SEG and prothoracic ganglion was cut, the crickets exhibited intact-like walking. The test crickets in which the left-side connective between the SEG and prothoracic ganglion was cut could walk with a tripod gait (*N* = 5, Figure 5, Supplementary Video 6 0:00-1:15). The crickets showed voluntary evoked walking. We focused on the periods of continuously walking to analyze the gait pattern. The phase differences between the left and right legs occurred in an anti-phase manner (Figures 5Aa–c). The foreleg and midleg of the same side moved in an anti-phase manner, whereas the foreleg and hindleg of the same side moved in an in-phase manner (Figures 5Ad–i). In the test crickets, the mean of the midleg phase difference Φ_*LM*−*RM*_ was 165°, with a standard deviation ν_*LM*−*RM*_ of 37.3°. The mean vector length *R*_*LM*−*RM*_ was 0.81. The shape of the empirical distribution function of the pair of midlegs of the test crickets was similar to that of the intact crickets (LM-RM: *n* = 109, Kuiper statistic vs. intact LM-RM:0.23 (*P* = 0.56), V statistic for 180°: 9.53 (*P* < 0.01), Table 1E, Figure 5B). Neither leg frequency nor stroke angle was significantly different from that of intact crickets under this experimental condition (Supplementary Figure 1D). We also examined the behavior when only the right-side connective between the SEG and prothoracic ganglion was cut. The results were quite similar to those of the crickets with the left-side connective cut (Supplementary Video 6 1:15–2:12).

**Figure 6:**
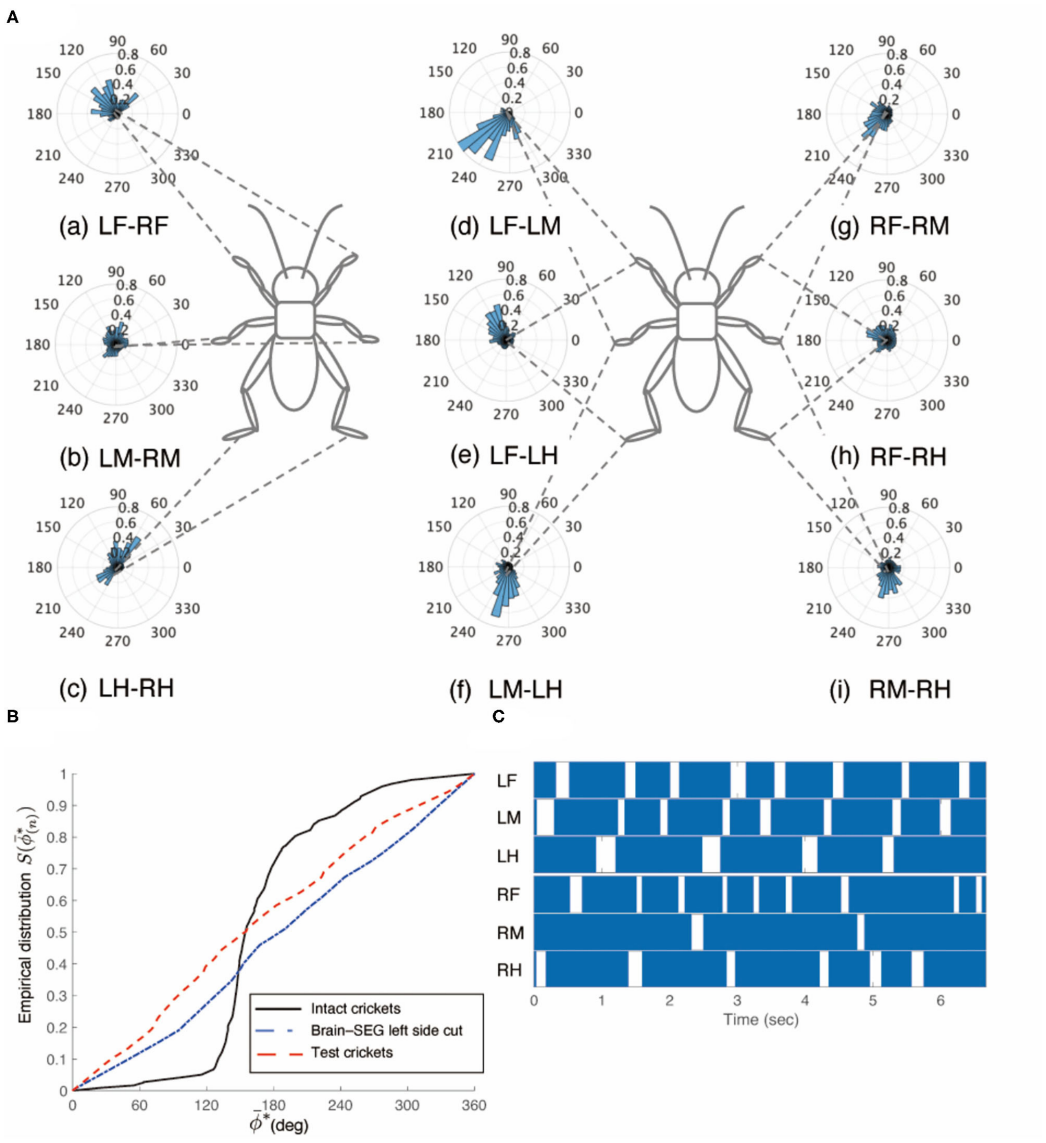
Walking gait patterns of crickets in which left sides of nerve connectives between brain and SEG, and between SEG and prothoracic ganglion were cut. **(A)** Polar histograms indicate phase differences between two legs in test crickets (*N* = 5). The test crickets continued to turn clockwise. The polar histograms demonstrate that the walking pattern was not a tripod gait. (a–i) Phase differences between pairs of legs. **(B)** Comparison of the empirical distribution function of phase differences between left and right legs Φ_*LM*−*RM*_. The black line indicates the empirical distribution function of the intact crickets; the blue line indicates that of the test crickets, and the red line indicates that of the crickets in which the left side of the nerve connectives between the brain and SEG was cut (shown in Figure 3B). **(C)** Gait chart diagram of test cricket. The filled parts indicate the duration of the power stroke period, and the blank part indicates the duration of the recovery stroke. This demonstrates that the walking pattern was not a tripod in the test crickets. LF, left foreleg; RF, right foreleg; LM, left midleg; RM, right midleg; LH, left hind leg; RH, right hind leg.

The crickets in which the left-side connectives between both the brain and SEG, and the SEG and prothoracic ganglion were cut did not walk straight forward, but continued to turn clockwise (*N* = 5, Supplementary Video 7 0:00–1:06). The walking of the crickets was evoked without tactile stimuli. We focused on the periods of continuously walking to analyze the gait pattern. The gaits in these test crickets did not exhibit an ordinary tripod pattern (Figure 6). The polar histogram of the test crickets indicates that the phase differences between the left and right legs were not consistent (Figure 6A). In the test crickets, the mean of the midleg phase difference Φ_*LM*−*RM*_ was 109°, with a standard deviation ν_*LM*−*RM*_ of 108°. The mean vector length *R*_*LM*−*RM*_ was 0.17. The shape of the empirical distribution function of the midlegs of the test crickets was different from that of the intact crickets [LM-RM: *n* = 121, Kuiper statistic vs. intact LM-RM: 0.59 (*P* < 0.01), V statistic for 180°: −1.38 (*P* = 0.92), Table 1F, Figure 6B]. The gait chart diagram of the test crickets demonstrates that the duration of the left leg movements appeared to be rhythmic, as in the intact crickets (Figure 6C). Compared with intact crickets, the frequencies of leg movements were rather low. In addition, the frequencies of the movement in the midleg and hind legs on the right side were smaller than those on the left side (Supplementary Figure 1E). The duration of the right legs touching the floor was much longer than that of the left legs. The angular stroke of the left midleg was not significantly different from that of intact crickets, while the angular stroke of the right midleg was suppressed. As a result, the test crickets turned in the clockwise direction. We also investigated the behavior of the crickets in which the right-side connectives between both the brain and SEG, and between the SEG and prothoracic ganglion were cut. These crickets continued to turn counterclockwise and did not exhibit a tripod gait (Supplementary Video 7 1:06–2:12).

**Figure 7:**
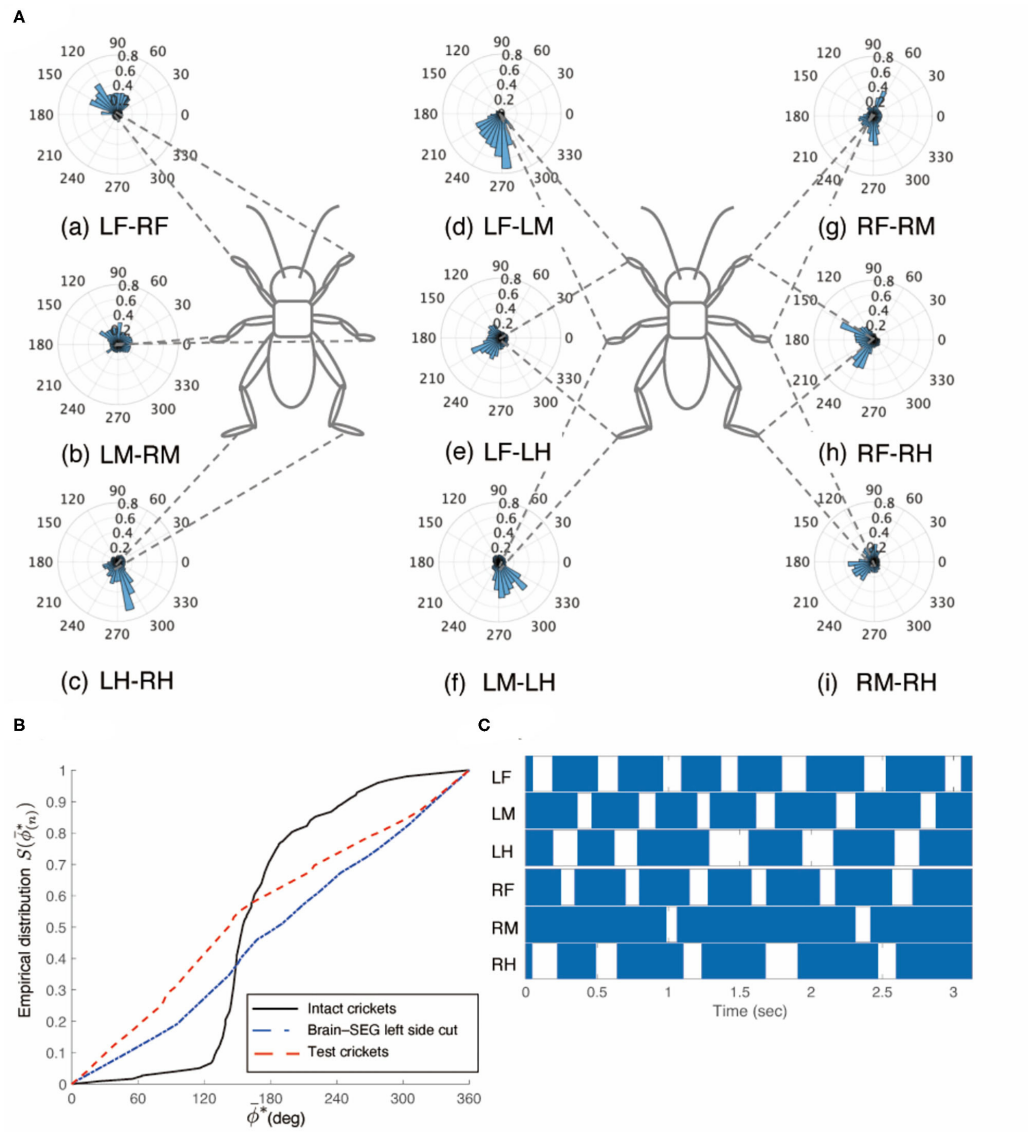
Walking gait patterns of crickets in which left side of nerve connectives between brain and SEG, and right side of connective between SEG and prothoracic ganglion were cut. **(A)** Polar histograms indicate phase differences between two legs in test crickets (*N* = 5). The test crickets continued to turn clockwise. The polar histograms demonstrate that the walking pattern was not a tripod gait. (a–i) Phase differences between pairs of legs. **(B)** Comparison of the empirical distribution function of phase differences between left and right legs Φ_*LM*−*RM*_ The black line indicates the empirical distribution function of the intact crickets; the blue line indicates that of the test crickets, and the red line indicates that of the crickets in which the left side of the nerve connectives between the brain and SEG was cut (shown in Figure 3B). **(C)** Gait chart diagram of test cricket. The filled parts indicate the duration of the power stroke period, and the blank part indicates the duration of the recovery stroke. This demonstrates that the walking pattern was not a tripod gait in the test crickets. LF, left foreleg; RF, right foreleg; LM, left midleg; RM, right midleg; LH, left hind leg; RH, right hind leg.

The behavior of the crickets in which the left-side connective between the brain and SEG, and the right-side connective between the SEG and prothoracic ganglion were cut was the same as that of the crickets in which the left-side connectives between the brain and the SEG, and between the SEG and prothoracic ganglion were cut. The walking of the crickets was evoked without tactile stimuli. Again, the test crickets did not walk straight forward, but continued to turn clockwise (*N* = 5, Supplementary Video 8 0:00–0:55). The gaits pattern of these test crickets did not exhibit a tripod (Figure 7). The polar histogram of the test crickets indicates that the phase differences between the left and right legs were not consistent (Figure 7A). In the test crickets, the mean of the midleg phase difference Φ_*LM*−*RM*_ was 87.5°, with a standard deviation ν_*LM*−*RM*_ of 110°. The mean vector length *R*_*LM*−*RM*_ was 0.16. The shape of the empirical distribution function of the midlegs of the test crickets was different from that of the intact crickets [LM-RM: *n* = 63, Kuiper statistic vs. intact LM-RM:0.58 (*P* < 0.01), V statistic for 180°: −3.46 (*P* = 1.00), Table 1G, Figure 7B]. The gait chart diagram of the test crickets demonstrates that the duration of the left leg movements appeared to be rhythmic, as in the intact crickets, but the right legs were not coordinated (Figure 7C). Compare to intact crickets, the frequencies of the leg movements were rather low. In addition, the frequencies of the leg movement on the right side were smaller than those on the left side (Supplementary Figure 1F). The angular stroke of the left midleg was not significantly different from that of intact crickets, while the angular stroke of the right midleg was suppressed. As a result, the test crickets turned in the clockwise direction. When the right-side connective between the brain and SEG, and the left-side connective between the SEG and prothoracic ganglion were cut, the test crickets continued to turn counterclockwise (Supplementary Video 8 0:55–2:12).

### Disconnection of Connectives Between Metathoracic Ganglion and Abdominal Ganglia

**Figure 8:**
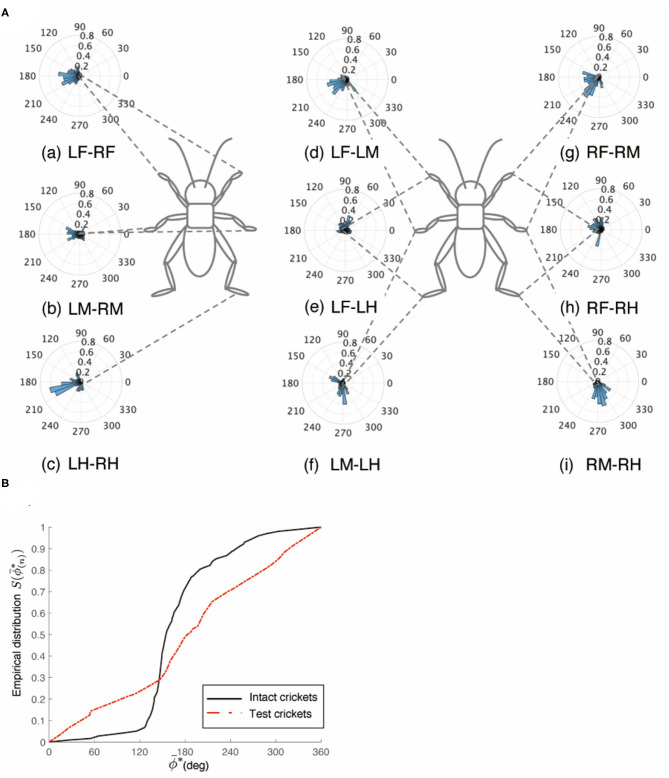
Walking gait patterns of crickets in which paired nerve connectives between metathoracic ganglion and abdominal ganglia were cut. **(A)** Polar histograms indicate phase differences between two legs in test crickets (*N* = 5). The polar histograms demonstrate that the test crickets did not exhibit a tripod gait pattern on the floating ball of the treadmill. (a–i) Phase differences between pairs of legs. **(B)** Comparison of the empirical distribution function of phase differences between left and right legs Φ_*LM*−*RM*_. The black line indicates the empirical distribution function of the intact crickets and the red line indicates that of the crickets in which the paired nerve connectives between the metathoracic ganglion and abdominal ganglia were cut. LF, left foreleg; RF, right foreleg; LM, left midleg; RM, right midleg; LH, left hind leg; RH, right hind leg.

**Figure 9:**
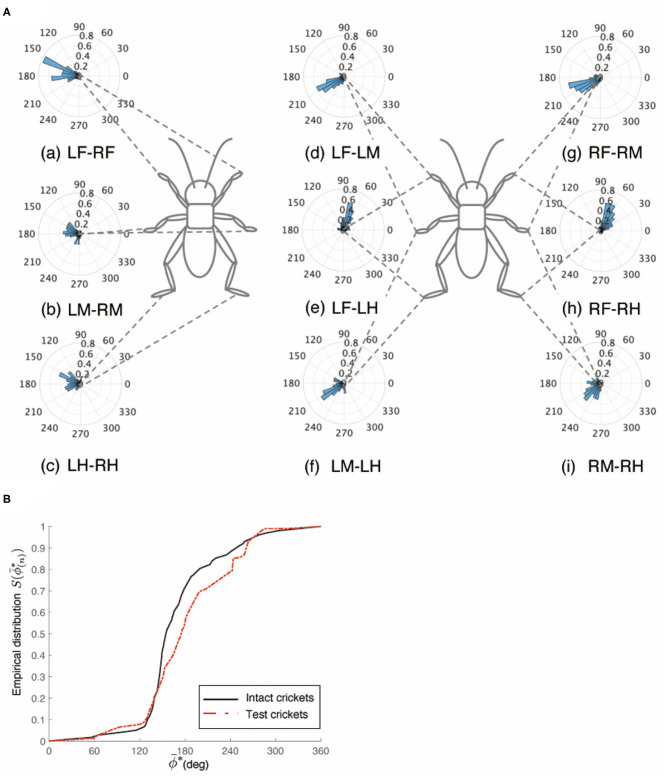
Walking gait patterns of crickets in which left side of nerve connectives between metathoracic ganglion and abdominal ganglia was cut. **(A)** Polar histograms indicate phase differences between two legs in test crickets (*N* = 5). The polar histograms demonstrate that the test crickets exhibited a tripod gait pattern on the floating ball of the treadmill. (a–i) Phase differences between pairs of legs. **(B)** Comparison of the empirical distribution function of phase differences between left and right legs Φ_*LM*−*RM*_. The black line indicates the empirical distribution function of the intact crickets and the red line indicates that of the crickets in which the left side of the nerve connectives between the metathoracic ganglion and abdominal ganglia was cut. LF, left foreleg; RF, right foreleg; LM, left midleg; RM, right midleg; LH, left hind leg; RH, right hind leg.

The crickets in which the pair of connectives between the metathoracic ganglion and first free abdominal ganglion was cut did not exhibit a tripod gait (*N* = 5, Figure 8A, Supplementary Video 9). The crickets showed voluntarily evoked walking although they did not respond with walking to the tactile stimuli of the cercus. The phase differences between the left and right midlegs were not consistent (Figure 8Aa). In the test crickets, the mean of the midleg phase difference Φ_*LM*−*RM*_ was 199°, with a standard deviation ν_*LM*−*RM*_ of 108°. The mean vector length *R*_*LM*−*RM*_ was 0.17. The shape of the empirical distribution function of the midlegs of the test crickets was far from that of the intact crickets [LM-RM: *n* = 71, Kuiper statistic vs. intact LM-RM:0.41 (*P* < 0.01), V statistic for 180°: 1.65 (*P* = 0.05), Table 1H, Figure 8B]. The frequency of the movements in all legs and the amplitude of the movement in the midleg were slightly lower than those of the intact cricket (Supplementary Figure 1G). However, the walking gait pattern in the crickets in which the left-side connective between the metathoracic ganglion and first free abdominal ganglion was cut exhibited an ordinary tripod gait pattern (Figure 9A, Supplementary Video 10 0:00–1:06). The walking was evoked voluntarily. The polar histogram of the test crickets in which the left-side connective was cut indicates that the phase differences between the left and right legs occurred in an anti-phase manner (Figure 9Aa). In the test crickets, the mean of the midleg phase difference Φ_*LM*−*RM*_ was 180°, with a standard deviation ν_*LM*−*RM*_ of 55.7°. The mean vector length *R*_*LM*−*RM*_ was 0.62. The shape of the empirical distribution function of the midlegs of the test crickets was similar to that of the intact crickets [LM-RM: *n* = 51, Kuiper statistic vs. intact LM-RM:0.23 (*P* = 0.56), V statistic for 180°: 4.12 (*P* < 0.01), Table 1I, Figure 9B]. The frequency of movements in all legs was slightly lower than those of the intact cricket (Supplementary Figure 1H). Similarly, when the right-side connective between the metathoracic ganglion and third abdominal ganglion was cut, the test crickets exhibited an intact-like tripod gait walk (Supplementary Video 10 1:06–2:20).

## Discussion

Crickets walk with a tripod gait pattern on a flat floor. Although the tripod gait is typical in insect walking, the gait patterns are not always fixed, but rather, change flexibly depending on the ground surface structure. The walking gait patterns may also vary if the body structure is changed; for example, owing to a loss of legs as a result of an accident (Full and Tu, 1991; Owaki et al., 2021). To evaluate the changes in the gait patterns, gait chart diagrams of insects have been drawn in many previous studies (Wilson, 1966). A gait chart diagram has also been used to evaluate the gait pattern of legged robots (Owaki et al., 2017). Such a diagram expresses the movements of each leg and clearly indicates a snapshot of the position of each leg. Moreover, polar histograms describing gait patterns evaluate the phase differences of a given pair of legs during walking (Naniwa et al., 2020). One of the advantages of using a polar histogram is that it enables us to evaluate the gait patterns of not only individuals but also a group of animals and legged robots. We investigated the effects of the loss of either the descending signals or ascending signals into the thoracic ganglia on regulating the cricket gait pattern.

### Descending Signals Into Thoracic Ganglia to Initiate Walking

Although the crickets in which the paired circumesophageal connectives were cut could walk, they did not change their direction while walking on the floating ball. The polar histograms of the treated crickets demonstrated that their gait was very close to the typical tripod pattern of the intact crickets in terms of inter-leg phase difference. As voluntary walking is initiated by descending signals originating in the brain (Kien and Altman, 1992; Kagaya and Takahata, 2011), the walking of the treated crickets was different from voluntary walking but could be initiated by receiving exteroceptive stimuli. The crickets responded to either tactile stimuli or air puffing on the cerci while walking. Crickets detect air currents using filiform hairs that are arranged on the surface of the cerci of the abdomen and respond with rapid avoidance movement when they are deflected (Edwards and Palka, 1974). Information on air movements is processed and integrated into the terminal abdominal ganglion, and the signals are transferred to the thoracic ganglia to initiate avoidance walking (Mendenhall and Murphey, 1974; Aonuma et al., 2008; Yono and Aonuma, 2008). Furthermore, the ascending signals from the abdominal nervous system also contribute to the initiation of walking; for example, after-defecation walking (Naniwa et al., 2019). Thus, certain types of internal or external stimuli contribute to the initiation of walking in brainless crickets. It has been reported that the brain inhibits all reflex activities (Bethe, 1898). Neuronal signals for coordinating the leg movements are generated in the thoracic ganglia of insects. A decrease in the inhibition from the brain may have contributed to the treated crickets walking straight forward in this study.

One of the remarkable findings of this study is that the crickets in which one side of the circumesophageal connectives was cut exhibited walking, while it continued to turn in the opposite direction to that of the surgical cut of the connective (Figure 3). The disconnection of the connective induces loss of frequency entrainment, which in turn causes loss of phase entrainment. This phenomenon appeared as though the inhibition from the brain to the cut-side pathway was abolished. The legs on the side of the connective cut moved more (Supplementary Figures 1C–F), which in turn pushed the body to the opposite side to continue turning. Movements of the opposite side could be introduced when they were bent. Therefore, the movements of the opposite side legs appeared to be caused by the local reflex. Movements of the legs in insects are detected by proprioceptive receptors (Tuthill and Wilson, 2016) such as the chordotonal organs (Hofmann et al., 1985; Büschges, 1994), campaniform sensilla (Bässler, 1977), and hair plate (Pearson et al., 1976; Wong and Pearson, 1976). Moreover, sensory afferents directly activate the extensor motor neurons of the trochanter and directly inhibit the flexor motor neurons in the cockroach (Pearson et al., 1976). The leg reflection initiated by a tactile stimulus was suppressed by the inhibitory descending signals from the brain, whereas the reflection occurs without brain signals in cockroach (Mu and Ritzmann, 2008). Thus, bending the leg joints could activate the directory extensor motor neurons to extend the legs of the crickets. Therefore, our results suggest that inhibition of the brain contributes to the regulation of coordinated walking in crickets.

The descending signals from the SEG into the thoracic ganglia are important for initiating walking. Inhibiting or blocking descending signals from SEG reduces the induction and maintenance of walking (Gal and Libersat, 2006, 2008). The crickets in which the paired connectives between the SEG and prothoracic ganglion were cut did not walk, except after defecation, as reported for the behavior of the headless cricket (Naniwa et al., 2019). The motor neurons that activate the leg muscles originate in the thoracic ganglia. The rhythmic activities of neurons, known as CPGs, in the thoracic ganglia are thought to be closely linked to coordinated leg movements (Büschges et al., 1995; Büschges, 1998; Ritzmann and Büschges, 2007). The CPGs are modulated by the descending signals from the brain that initiate, maintain, modify, and stop the motor outputs for walking (Bidaye et al., 2017). The roles of the SEG are believed to modulate the interactions between the sensory inputs from the legs and motor output (Knebel et al., 2018, 2019). It has also been reported that descending signals from the SEG can exhibit pattern generators in the chest and abdomen (Kien, 1990). It has been reported that the SEG plays an important role in the initiation, maintenance, and coordination of walking in the locust (Kien and Altman, 1984). Our behavior experiments confirmed the important role of the SEG in initiating walking.

Another significant finding in this study is that the crickets in which one side of the paired connective between the SEG and prothoracic ganglion was cut walked like the intact crickets (Figures 5, 9). Furthermore, the crickets in which one side of the circumesophageal connectives and one side of the connectives between either the ipsilateral or contralateral side of the SEG and prothoracic ganglion were cut continued to turn in the opposite side to that of the circumesophageal connective cut (Figures 6, 7). This suggests that the descending signals from the SEG converge and are processed in the thoracic ganglia and that the leg movements are regulated by the information from the SEG, even if it is only passed through one side of the connectives. Therefore, neurons may exist that integrate the information passed through the left and right pathways. Bilaterally symmetrical dorsal unpaired median (DUM) neurons have been identified in insects [locust: (Plotnikova, 1969); cockroach: (Crossman et al., 1971); and crickets (Clark, 1976)]. Certain DUM neurons terminate in the leg muscles of cockroaches (Denburg and Barker, 1982; Tanaka and Washio, 1988). Moreover, the DUM neurons in the prothoracic ganglion contribute to walking regulation in crickets (Gras et al., 1990). In the case of locusts, the effect of the neural network comprising the brain, subesophageal ganglion, and thoracic ganglion on locomotion patterns has been investigated (Kien, 1983; Kien and Williams, 1983). Further investigation is required to clarify which neurons contribute to interlimb coordination in crickets.

### Effect of Ascending Signals From Abdominal Nervous System on Walking

In insects, the abdominal nervous system serves as the center for controlling avoidance behavior (Mendenhall and Murphey, 1974; Tauber and Camhi, 1995; Card, 2012), mating behavior (Killian et al., 2006), egg-laying behavior (Sugawara and Loher, 1986), and defecation walking (Naniwa et al., 2019). These behaviors are closely linked to walking. Therefore, ascending signals from the abdominal ganglia into the thoracic ganglia may contribute to initiating and regulating walking in crickets. Furthermore, the descending signals that are modulated by the sensory feedback signals from the legs contribute to the modulation of the coordinated walking gait (Bidaye et al., 2017; Knebel et al., 2018). Thoracic ganglia form a network as CPGs that are spontaneously excited by SEG to establish a constant rhythm, while also coordinating leg motor patterns based on ascending signals from the lower ganglia (Bässler et al., 1985; Kien and Altman, 1992; Knebel et al., 2019). The coordinated rhythmic leg motor pattern is modulated by sensory signals acquired by mechanoreceptive organs of the legs (Owaki et al., 2021). These studies indicate the activities of the CPGs in the thoracic ganglia are modulated by multi kinds of signals, e.g., descending signals, sensory feedback, and so on. Our results add ascending signals as other signals to modulate CPG activities in the thoracic ganglia. Activation of the giant interneurons originated in the terminal abdominal ganglion elicit avoidance walking in the crickets (e.g., Jacobs and Murphey, 1987; Yono and Aonuma, 2008). Some of the giant neurons innervate axons into the thoracic ganglia and extend neuronal branches (Hirota et al., 1993). The neuronal branches of the ascending neurons in the anterior ganglia have outputs to motor control (Aonuma et al., 1994). Therefore, ascending signals from the abdominal nervous systems could modulate motor control in the thoracic ganglia. Ascending signals from the abdominal nervous systems and the descending signals from the brain and SEG could converge in the thoracic ganglia to coordinate walking gait patterns in crickets. The disconnection of the paired connectives between the metathoracic ganglion and first free abdominal ganglion prevented tripod gait walking in the crickets. However, the disconnection of one side of the connectives between the metathoracic ganglion and first free abdominal ganglion did not affect the expression of the tripod gait. Therefore, similar to the descending signals from the SEG into the thoracic ganglia, the ascending signals may be transferred into the bilateral neurons to be integrated and processed in the thoracic ganglia. Coordinated walking gait patterns are thought to be produced by the CPGs, descending central commands, and sensory feedback loops. This study demonstrated that the ascending signals from the abdominal nervous system also contribute to the generation of coordinated walking gait patterns in insects. It is technically difficult to cut the metathoracic ganglion and to fuse the first and second abdominal ganglia to examine how these ganglia contribute to the coordinated gait pattern. In contrast, cutting between the terminal abdominal ganglion and the sixth abdominal ganglion did not affect the expression of the tripod gait pattern (Supplementary Video 11). This suggests that the sensory signals from cercus may not mainly contribute to the expression of the tripod pattern. Thus, it is necessary to investigate which ganglion or which group of ganglia interact with SEG to coordinate the tripod gait and to investigate which types of neurons contribute to regulating the leg movements in crickets.

## Data Availability Statement

The original contributions presented in the study are included in the article/Supplementary Material, further inquiries can be directed to the corresponding author/s.

## Author Contributions

KN and HA conceived and designed the experiment, performed the experiment, analyzed the data, and wrote the manuscript.

## Conflict of Interest

The authors declare that the research was conducted in the absence of any commercial or financial relationships that could be construed as a potential conflict of interest.

## Supplementary Material

Supplementary Figure 1 Mean and standard deviation of the frequency of movement of each leg and the movement amplitude of the midleg. The black letters indicate the frequencies of the leg movements. The red letters indicate the stroke angle of the midleg. A comparison test between intact cricket and experimental crickets was performed. A comparison test between the right and left middle leg in the experimental cricket was performed. Mann–Whitney *U*-test was used for the comparison test of the leg frequency and the movement amplitude. Significance level α = 0.01. **(A)** Intact crickets exhibited a tripod gait. **(B)** Disconnection of the paired circumesophageal exhibited a tripod gait. **(C)** Disconnection of the left-side of the circumesophageal connective exhibited a turn clockwise. **(D)** Disconnection of the left-side connective between the SEG and prothoracic ganglion exhibited a tripod gait. **(E)** Disconnection of the left-side of the circumesophageal connective and the left-side connective between the SEG and prothoracic ganglion (cutting ipsilateral side) exhibited a turn clockwise. **(F)** Disconnection of the left-side of the circumesophageal connective and the right-side connective between the SEG and prothoracic ganglion exhibited a turn clockwise. **(G)** Disconnection of the paired connectives between the metathoracic ganglion and first free abdominal ganglion exhibited an uncoordinated gait. **(H)** Disconnection of the left-side connective between the metathoracic ganglion and first free abdominal ganglion exhibited a tripod gait.
