# Supplementary figures and images for: Descending and Ascending Signals That Maintain Rhythmic Walking Pattern in Crickets
Source: Front Robot AI. 2021 Mar 29;8:625094. doi: 10.3389/frobt.2021.625094 (PMC8039156; doi:10.3389/frobt.2021.625094)

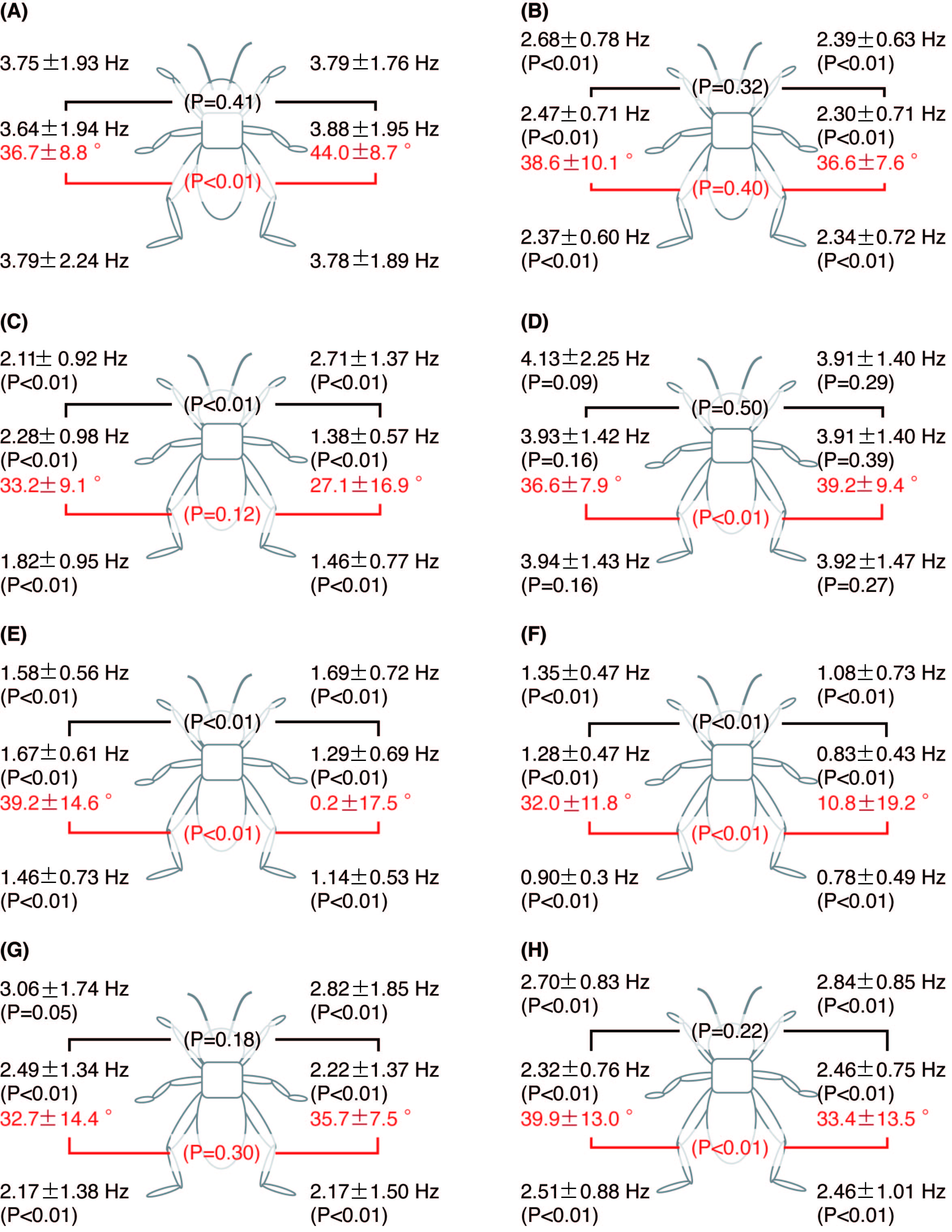

Supplement: Supplementary Figure 1 — Mean and standard deviation of the frequency of movement of each leg and the movement amplitude of the midleg. The black letters indicate the frequencies of the leg movements. The red letters indicate the stroke angle of the midleg. A comparison test between intact cricket and experimental crickets was performed. A comparison test between the right and left middle leg in the experimental cricket was performed. Mann–Whitney U-test was used for the comparison test of the leg frequency and the movement amplitude. Significance level α = 0.01. (A) Intact crickets exhibited a tripod gait. (B) Disconnection of the paired circumesophageal exhibited a tripod gait. (C) Disconnection of the left-side of the circumesophageal connective exhibited a turn clockwise. (D) Disconnection of the left-side connective between the SEG and prothoracic ganglion exhibited a tripod gait. (E) Disconnection of the left-side of the circumesophageal connective and the left-side connective between the SEG and prothoracic ganglion (cutting ipsilateral side) exhibited a turn clockwise. (F) Disconnection of the left-side of the circumesophageal connective and the right-side connective between the SEG and prothoracic ganglion exhibited a turn clockwise. (G) Disconnection of the paired connectives between the metathoracic ganglion and first free abdominal ganglion exhibited an uncoordinated gait. (H) Disconnection of the left-side connective between the metathoracic ganglion and first free abdominal ganglion exhibited a tripod gait. [file Image_1.JPEG]
